# Effect of divalent ions and a polyphosphate on composition, structure, and stiffness of simulated drinking water biofilms

**DOI:** 10.1038/s41522-018-0058-1

**Published:** 2018-07-18

**Authors:** Yun Shen, Pin Chieh Huang, Conghui Huang, Peng Sun, Guillermo L. Monroy, Wenjing Wu, Jie Lin, Rosa M. Espinosa-Marzal, Stephen A. Boppart, Wen-Tso Liu, Thanh H. Nguyen

**Affiliations:** 1 Department of Civil and Environmental Engineering, University of Illinois at Urbana–Champaign, Urbana, IL USA; 2 Department of Bioengineering, University of Illinois at Urbana–Champaign, Urbana, IL USA; 3 Department of Electrical and Computer Engineering, University of Illinois at Urbana-Champaign, Urbana, IL USA; 4 Present Address: University of Michigan, 1351 Beal Ave., 219 EWRE Bldg, Ann Arbor, MI 48109-2125 USA

## Abstract

The biofilm chemical and physical properties in engineered systems play an important role in governing pathogen transmission, fouling facilities, and corroding metal surfaces. Here, we investigated how simulated drinking water biofilm chemical composition, structure, and stiffness responded to the common scale control practice of adjusting divalent ions and adding polyphosphate. Magnetomotive optical coherence elastography (MM-OCE), a tool developed for diagnosing diseased tissues, was used to determine biofilm stiffness in this study. MM-OCE, together with atomic force microscopy (AFM), revealed that the biofilms developed from a drinking water source with high divalent ions were stiffer compared to biofilms developed either from the drinking water source with low divalent ions or the water containing a scale inhibitor (a polyphosphate). The higher stiffness of biofilms developed from the water containing high divalent ions was attributed to the high content of calcium carbonate, suggested by biofilm composition examination. In addition, by examining the biofilm structure using optical coherence tomography (OCT), the highest biofilm thickness was found for biofilms developed from the water containing the polyphosphate. Compared to the stiff biofilms developed from the water containing high divalent ions, the soft and thick biofilms developed from the water containing polyphosphate will be expected to have higher detachment under drinking water flow. This study suggested that water chemistry could be used to predict the biofilm properties and subsequently design the microbial safety control strategies.

## Drinking water: Ions that influence biofilms

A variety of analytical techniques are revealing the complex influences of ions in drinking water supplies on the structure of biofilms. Such biofilms often contaminate water supply pipes and machinery. Yun Shen and colleagues at the University of Illinois at Urbana-Champaign in the USA investigated the effects of ions with a double positive charge – ‘divalent cations’ – and polyphosphate ions. Divalent cations, especially calcium and magnesium ions, are abundant in drinking water in many regions, promoting the formation of limescale deposits. Polyphosphates are commonly added to water supplies to reduce limescale formation, inhibit corrosion and discourage biofilm formation. The research revealed that divalent cations increase biofilm stiffness, while polyphosphates promote softer but thicker biofilms that are more easily removed. The results will help optimize water treatment procedures to control both microbial contamination and limescale problems.

## Introduction

Biofilms are ubiquitously found in engineered water systems, food industries, healthcare facilities, and medical devices. These biofilms, composed of extracellular biopolymers, microorganisms, and inorganic particles, facilitate pathogen transmission, foul the facilities and instruments, and accelerate metal surface corrosion.^1^ Specifically, biofilms on engineered surfaces, such as drinking water pipes, food contact surfaces, and even medical implant surfaces, are identified as a reservoir of pathogens.^2–6^ Biofilms can protect pathogens from environmental stresses (e.g., disinfectants, fluidic shearing, and low nutrients) and then release pathogens into drinking water, food, or human body through biofilm sloughing off.^7,8^ In addition, biofilms create fouling on water treatment facilities and food process lines, thus increasing head loss and energy cost.^1,9^ Furthermore, the metal parts in the engineered water systems can be damaged by biofilm-induced corrosion.^10^ Biofilm chemical and physical properties are responsible for the biofilm-associated pathogen transmission, fouling, and corrosion. For example, biofilm chemical composition affects the dissolution of cast iron surfaces.^11^ Biofilm physical structure, especially biofilm roughness, has been shown to enhance pathogen accumulation on biofilms and prevent pathogen release from biofilms.^12–14^ Biofilm stiffness controls the release of biofilm-associated pathogens and influences biofilm removal to mitigate fouling.^15,16^ Therefore, understanding the biofilm composition, structure, and stiffness will provide insights on pathogen control and engineered system maintenance.

However, the factors influencing biofilm chemical and physical properties are still largely unknown. The complex environmental matrices (e.g., temperature, pH, water chemistry, nutrients, etc.) can potentially impact the biofilm properties.^17,18^ In particular, divalent ions (such as Ca^2+^ and Mg^2+^), which are abundant in some food and water, may potentially affect the biofilm chemical and physical properties. Previous studies suggested that exposing pre-established biofilms to a high concentration of Ca^2+^ improved biofilm cohesiveness by cross-linking biopolymers and binding microorganisms together.^19,20^ Nevertheless, how continuous exposure of divalent ions during biofilm development affects biofilm properties is still unknown. In addition, the divalent ions form mineral deposits, which are also known as scales, in the engineered surfaces. These scales, sometimes coupled with and incorporated into biofilms, may further affect the biofilm structure and stiffness. However, as a possible biofilm component, how these deposits influence biofilm properties has been underexplored. Especially, scale control by reducing the Ca^2+^ and Mg^2+^ (e.g., ion exchange, chemical precipitation, or membrane processes) is a common practice applied in water treatment processes.^21^ Understanding how the divalent ions and scale deposits influence biofilm properties could help to assess the water microbial risk and optimize the water supply system design and management. In addition to reducing the divalent ions, a scale inhibitor has also been used in the engineered water systems to mitigate fouling caused by scale formation. Polyphosphate, a widely used scale inhibitor in engineered water systems, prevents excessive formation of calcium carbonate scales and in some cases inhibits corrosion from metal pipes and decolors the “red water”.^22–24^ Meanwhile, the phosphate species can support the growth of bacteria, thereby facilitating biofilm biomass accumulation.^25^ However, how the addition of polyphosphate influences biofilm chemical composition, structure, and stiffness is unknown.

Therefore, systematically studying the effect of divalent ions and polyphosphate on biofilm chemical composition, structure, and stiffness is necessary to fill the aforementioned research gaps. However, while the current technologies allow for the determination of biofilm structure and composition,^26–28^ determining biofilm stiffness remains challenging. With the aid of atomic force microscopy (AFM), the amyloid and protein components in *Pseudomonas* and *Lactobacillus* biofilms were suggested to increase the biofilm stiffness, respectively.^29,30^ While AFM was applied mainly to measure the stiffness of the biofilm near surface regions (depth up to 5 μm),^30–33^ rheometers and tensile tests were utilized to determine the biofilm bulk stiffness.^34–37^ By using a rheometer, the stiffness of *Pseudomonas aeruginosa* was found to significantly increase after exposing to multivalent ions (Ca^2+^, Fe^3+^, and Al^3+^).^37,38^ However, the rheometer or tensile methods required high biofilm thickness or destructive and tedious pre-handling processes (e.g., biofilm staining).^34–37^ In addition, quantitative understanding of the links between composition, structure, and stiffness of native biofilms developed under different environmental conditions is still lacking. Therefore, the evolution of the stiffness measurement techniques is needed, and incorporation of the biofilm stiffness measurement with other biofilm composition and structure examination methods is necessary to explore the links between biofilm physical and chemical properties.

This study showed that the chemical composition, structure, and stiffness of biofilms were strongly affected by divalent ions and the addition of polyphosphate. A local drinking water source (groundwater containing Ca^2+^ and Mg^2+^ as the main divalent ion sources) was used to develop biofilms in this study. To simulate the conditions under common scale control practices (reducing water hardness and adding a polyphosphate), biofilms were developed from untreated groundwater with a high divalent ion concentration (or high hardness), groundwater with reduced divalent ion concentration (or reduced hardness), and groundwater added with a polyphosphate (sodium hexametaphosphate (SHMP)), respectively. The chemical composition, structure, and stiffness of these three types of biofilms was determined by attenuated total reflection-Fourier transform infrared spectroscopy (ATR-FTIR) and thermogravimetric analysis (TGA), optical coherence tomography (OCT), and AFM. In addition, magnetomotive optical coherence elastography (MM-OCE), a novel method developed for medical diagnosis (e.g., to detect diseased tissues that are stiffer than normal tissues),^39–44^ was also used to measure the biofilm bulk stiffness. The novel and unique contributions in our study are as follows: (1) we identified, for the first time, the important role of two most common scale control practices on the physicochemical properties of multi-culture biofilms developed from a real drinking water source; (2) we developed methodologies to systematically study the biofilm physicochemical properties and apply the MM-OCE method for biofilm stiffness determination for the first time; and (3) our study revealed the link between biofilm chemical composition and biofilm stiffness. The results of this study can provide insights on prediction of biofilm properties and management of engineered water systems.

## Results

### Biofilm composition determined by FTIR and TGA

**Fig. 1:**
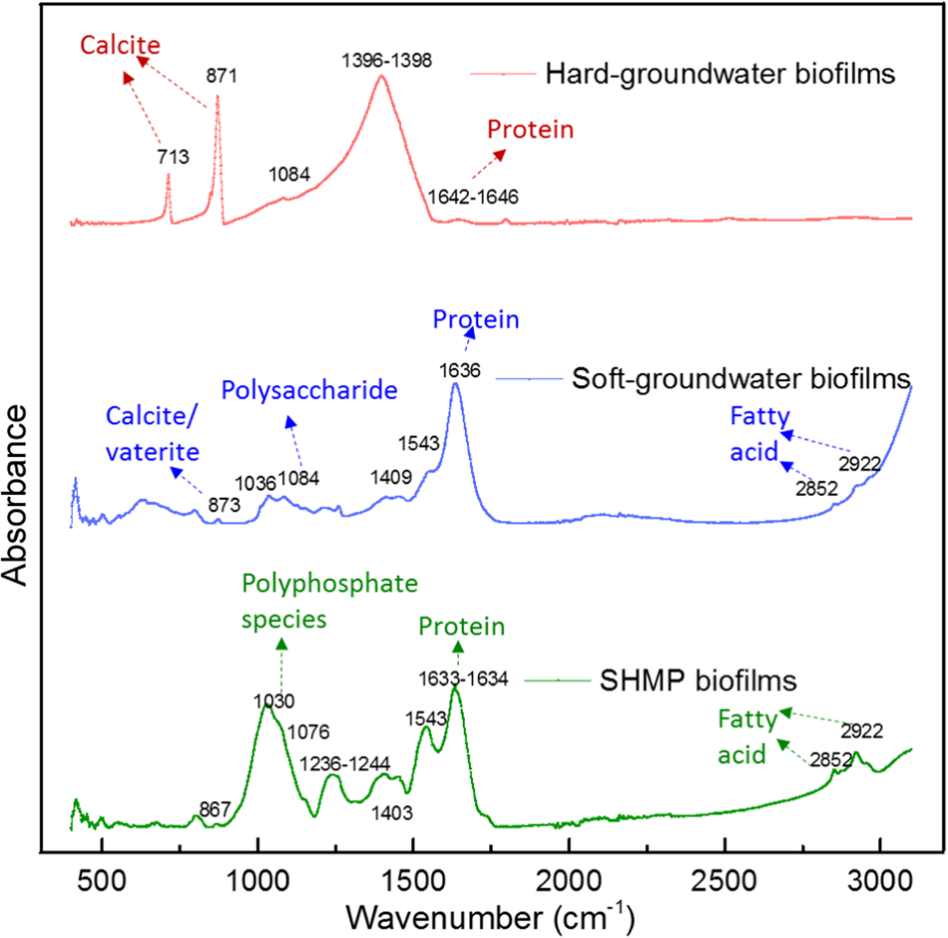
ATR-FTIR spectra of hard-groundwater biofilms, soft-groundwater biofilms, and SHMP biofilms. The main absorbance peaks are shown in the figure

The infrared (IR) spectrums obtained by ATR-FTIR for biofilms developed from raw groundwater with high hardness (hard groundwater), groundwater with reduced hardness (soft groundwater), and groundwater containing SHMP are shown in Fig. 1. For the hard-groundwater biofilms, the three highest peaks at 713 cm^−1^, 871 cm^−1^, and 1398 cm^−1^ in the spectra were observed. The peak at 713 cm^−1^ was representative of aragonite or calcite,^45^ revealing the existence of crystallized calcium carbonate (CaCO_3_) in hard-groundwater biofilms. The absorbance peak at 871 cm^−1^ was attributed to CaCO_3_ polymorphs. According to previous studies,^45,46^ 866 cm^−1^ represented either crystalline or amorphous calcium carbonate (ACC), while 876 cm^−1^ corresponded to calcite. Therefore, the 871 cm^−1^ peak may result from the presence of a crystalline form of CaCO_3_ or ACC. The 1398 cm^−1^ peak represented either carboxylate ions or carbonate.^28,45^ In addition to these three high-intensity peaks, very small peaks were observed at 1084 cm^−1^ and 1646 cm^−1^, and these two peaks may represent the ring structure of polysaccharide and unordered protein, respectively,^28,47^ as the main biomolecular components of a biofilm. However, their absorbance peaks in hard-groundwater biofilms were not very pronounced (e.g., the ratio of the calcite absorbance peak at 871 cm^−1^ to the protein absorbance peak at 1646 cm^−1^ was 17).

Unlike the IR spectrum of the hard-groundwater biofilm revealing a high content of CaCO_3_ species, the soft-groundwater biofilm spectrum showed high organic components. The highest peak observed at 1636 cm^−1^ represented the amide I group of some proteins (e.g., trypsinogen and carbonic anhydrase).^47^ A small peak detected at 873 cm^−1^, indicates that calcite may exist in the soft-groundwater biofilms. The ratio of the calcite (873 cm^−1^) to the protein (1636 cm^−1^) absorbance peak, 0.088, for the soft-groundwater biofilms was much lower compared with the ratio (17) for the hard-groundwater biofilms. The 1084 cm^−1^ peak attributed to polysaccharides was also detected. Furthermore, two peaks were detected at 2852 cm^−1^ and 2922 cm^−1^, corresponding to the fatty acid component in biofilms.^28^ In the spectrum of biofilms developed from groundwater containing SHMP (SHMP biofilms), the peaks attributed to fatty acid and protein were also detected. In addition, one high-intensity peak at 1030 cm^−1^ was detected, and has been previously reported for pure SHMP solutions.^48^ Thus, the absorbance peak at 1030 cm^−1^ revealed the presence of polyphosphate species in the SHMP biofilms. In conclusion, the ATR-FTIR measurement results indicated the abundance of crystalline CaCO_3_ polymorphs in the hard-groundwater biofilms, while the organic components dominated the soft-groundwater and SHMP biofilms.

**Fig. 2:**
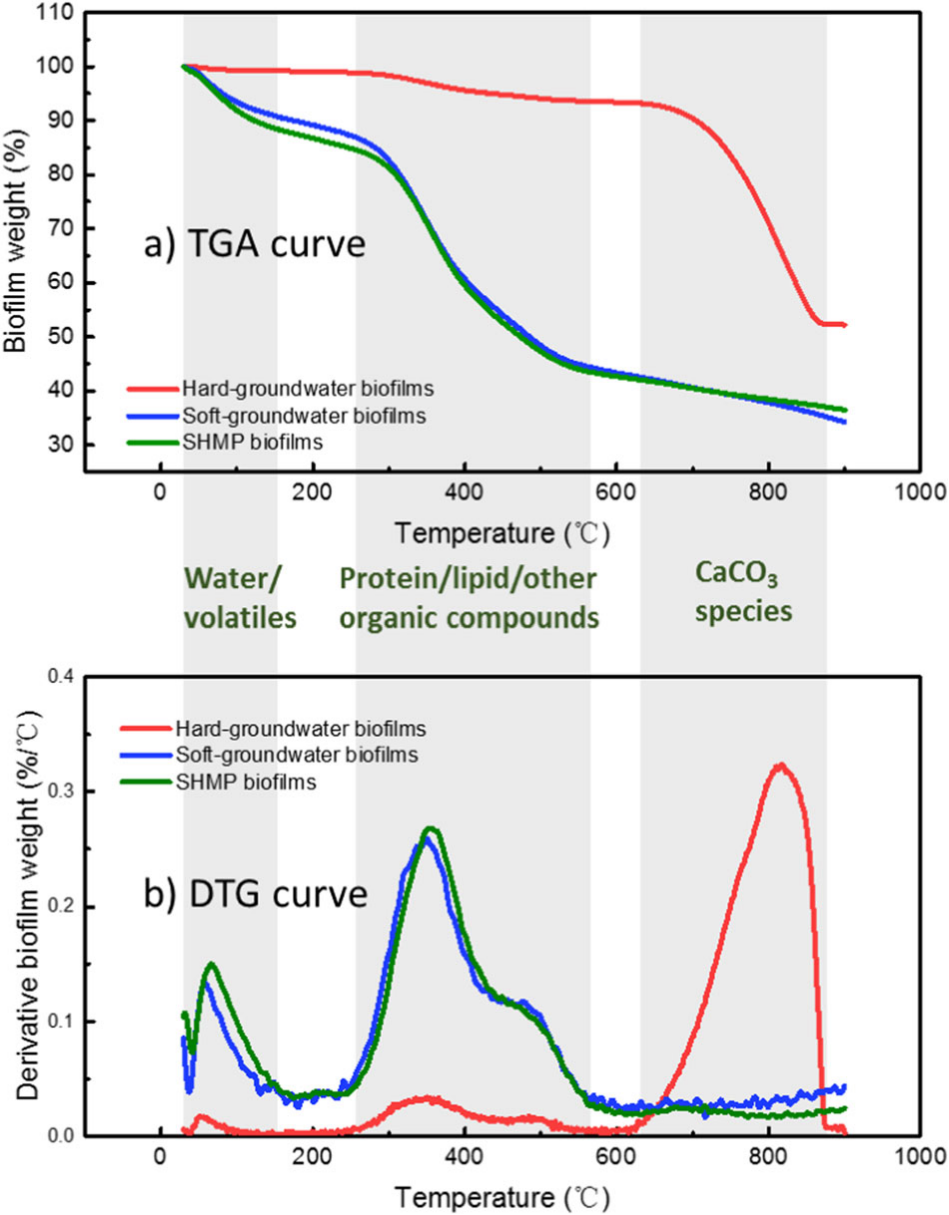
**a** TGA curve showing the percentage of biofilm weight at a certain temperature with respect to the initial biofilm weight as a function of temperature and **b** derivative thermogravimetric analysis (DTG) curve showing the change of biofilm decomposition rate as a function of temperature. The error bars represent the standard deviation of the measurements for the replicates

The weight percentage of inorganic components (e.g., CaCO_3_) and organic components (e.g., protein, polysaccharide, and lipid) in biofilms was determined by TGA. Figure 2a shows that the main weight loss of the soft-groundwater and SHMP biofilms occurred in the temperatures ranging from 250 °C to 560 °C; while in the hard-groundwater biofilms, the main weight loss occurred in the temperatures ranging from 620 °C to 810 °C. To more clearly show the biofilm thermal decomposition stages, the weight loss rate of biofilms was plotted with respect to temperature (derivative thermogravimetric or DTG curve, Fig. 2b). The DTG curves for soft-groundwater and SHMP biofilms showed similar profiles with two characteristic peaks (68 and 357 °C for the SHMP biofilms, and 60 and 351 °C for the soft-groundwater biofilms). Here, we take the DTG curve of SHMP biofilms as an example. The first peak (37–173 °C) in the DTG curve for SHMP biofilms corresponded to a 9.6% loss of biofilm weight, attributing to the loss of residual water and highly volatile compounds in biofilms.^49^ The second peak (232–559 °C) revealed 43.8% of biofilm weight loss. According to previous studies,^49,50^ microalgae biomass and the organic components in the biomass (e.g., lipids and protein) were thermally degraded mainly at the temperatures ranging from 200 °C to 600 °C. Since the biofilm biomass contains similar types of organic components with the microalgae biomass, these organic components or biopolymers (i.e., protein, carbohydrate, and lipid) in biofilms are expected to pyrolyze at a similar temperature range. Therefore, the second peak corresponded to the weight loss caused by the pyrolysis of biopolymers in biofilms. While the DTG curves of soft-groundwater and SHMP biofilms showed two peaks, the DTG curve of hard groundwater exhibited three peaks. The first peak throughout the temperature range of 36–102 °C revealed a weight loss (0.6%) of water and highly volatile compounds. A weight loss of biopolymers (5.5%) was revealed by the second peak (241–574 °C). The highest weight loss (40.8%) occurred at the third peak (624–877 °C), due to the pyrolysis of inorganic compounds (salts). The third peak observed in the hard-groundwater DTG curve was assigned to the calcination of precipitated CaCO_3_, according to the results obtained by ATR-FTIR and inductively coupled plasma mass spectrometry (ICP-MS, Supplementary Information). Specifically, ATR-FTIR revealed the presence of CaCO_3_ in hard-groundwater biofilms. The ICP-MS measurements showed a higher content of calcium in hard-groundwater biofilms (4.51 mg/biofilm coupon) than in soft-groundwater (0.01 mg/biofilm coupon) and SHMP biofilms (0.17 mg/biofilm coupon). Nevertheless, it is possible that organic components are embedded into the precipitated CaCO_3_; thus, their thermal decomposition might occur simultaneously to calcination.

### Biofilm structure

**Fig. 3:**
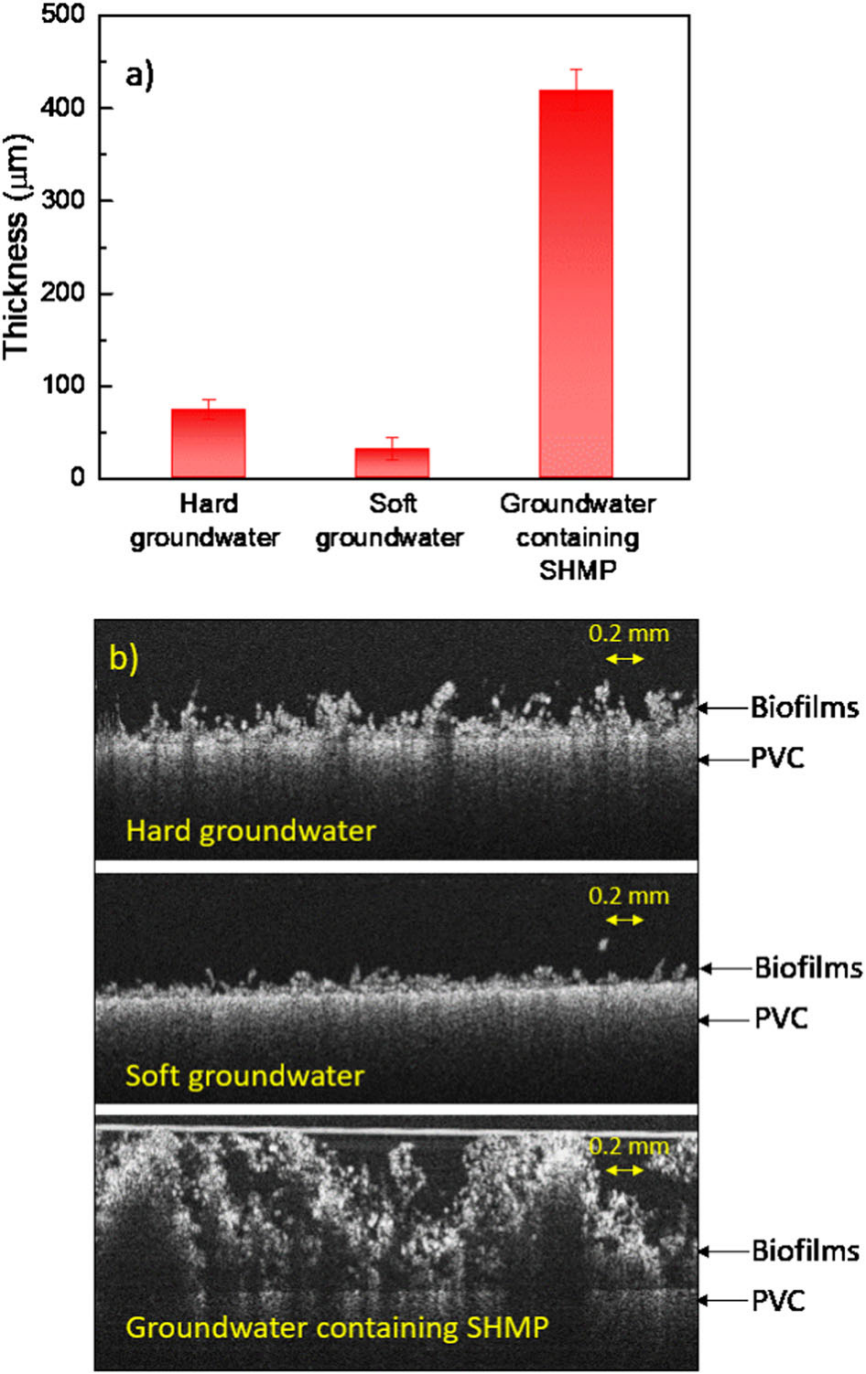
**a** Average thickness and **b** selected OCT images of biofilms developed from hard groundwater, soft groundwater, and groundwater containing SHMP

The average thickness of hard-groundwater biofilms, soft-groundwater biofilms, and SHMP biofilms was measured by OCT (Fig. 3). Among the three types of biofilms, the SHMP biofilms showed the highest thickness (418 ± 21 μm), which was 6–14 times thicker than the hard- and soft-groundwater biofilms. The addition of polyphosphate during biofilm development can supply nutrients to bacteria, take an important role in bacteria metabolic regulation, and assist bacteria to develop resistance to environmental stress.^25,51,52^ The soft-groundwater biofilms were the thinnest (30 ± 12 μm), probably because ion exchange resin treatment reduced the organic matter content in groundwater. Furthermore, the roughness of these three types of biofilms was analyzed (Figure S2). The soft-groundwater biofilms had the highest relative roughness coefficient (0.71 ± 0.15) and the lowest absolute roughness (21 ± 10 μm), while the SHMP biofilms had the lowest relative roughness coefficient of 0.19 ± 0.04 and the highest absolute roughness of 79 ± 18 μm.

### Biofilm stiffness

**Fig. 4:**
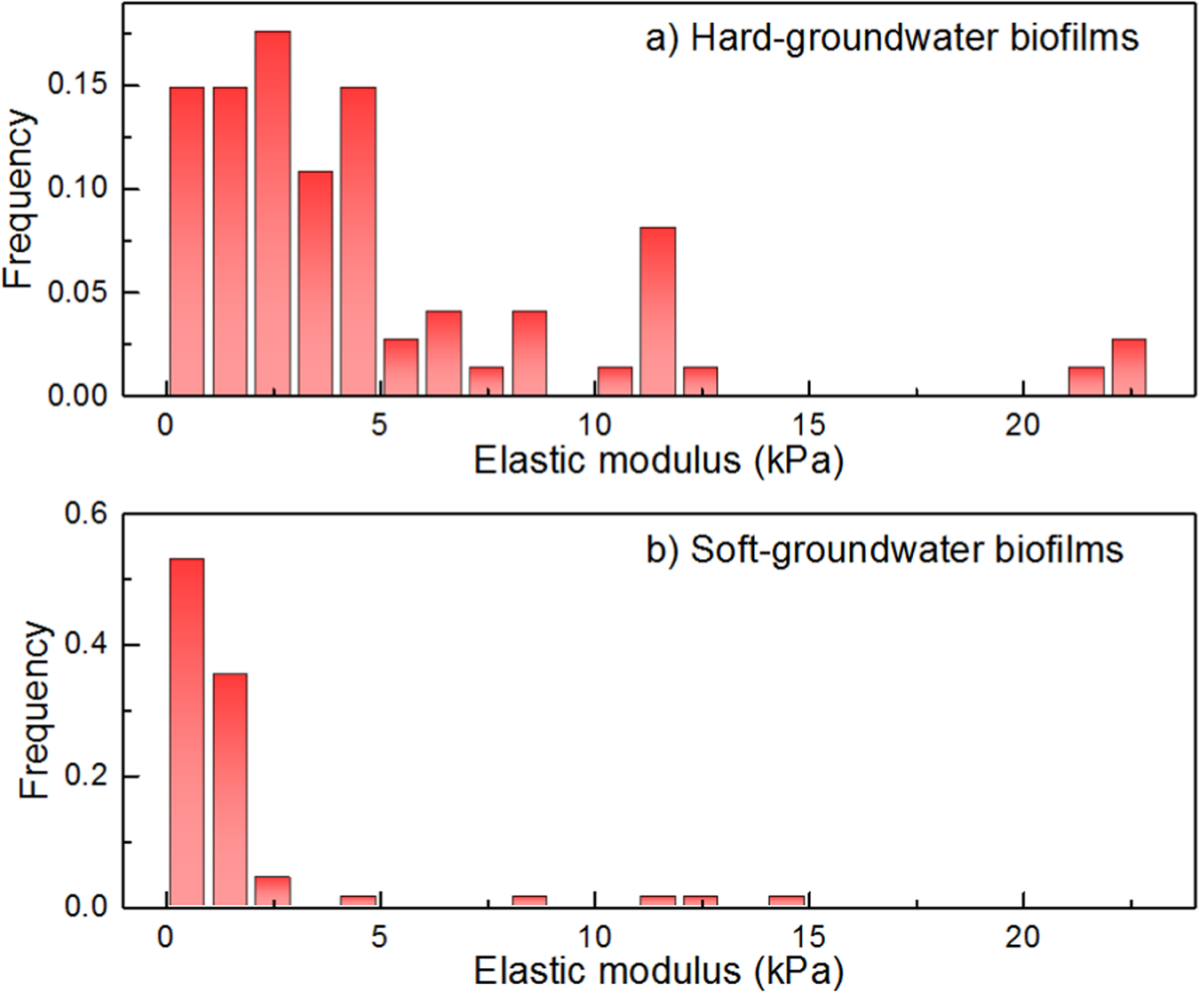
The frequency distributions of elastic modulus values for **a** the hard-groundwater biofilms and **b** soft-groundwater biofilms

To evaluate the near-surface stiffness of biofilms, the frequency distribution of the biofilm elastic modulus was determined by AFM indentation tests (Fig. 4a, b). The elastic modulus frequency distributions of hard- and soft-groundwater biofilms were statistically different (Kolmogorov–Smirnov test, *p* = 8 × 10^−12^). Compared with the soft-groundwater biofilms, the hard-groundwater biofilms had elastic modulus values distributed in a wider and higher value range. In particular, the highest frequency (18%) of the elastic modulus for hard-groundwater biofilms was observed at 3–4 kPa, while the highest frequency (53%) of the elastic modulus for soft-groundwater biofilms was found at 0–1 kPa. The results obtained by AFM indentation tests suggested that the hard-groundwater biofilms were stiffer than the soft-groundwater biofilms. However, AFM indentation tests only quantified the stiffness of the near-surface layer in the biofilms, while the overall biofilm stiffness was not detected. In addition, applying AFM indentation tests to very soft and flexible samples in the water phase is challenging. Here, we failed to utilize AFM to determine the near-surface elastic modulus for the SHMP biofilms, due to the high flexibility of SHMP biofilms when the biofilms were immersed in water. To determine the biofilm overall stiffness and compare the stiffness for all three types of biofilms used in this study, the MM-OCE method was performed and the results are discussed.

**Fig. 5:**
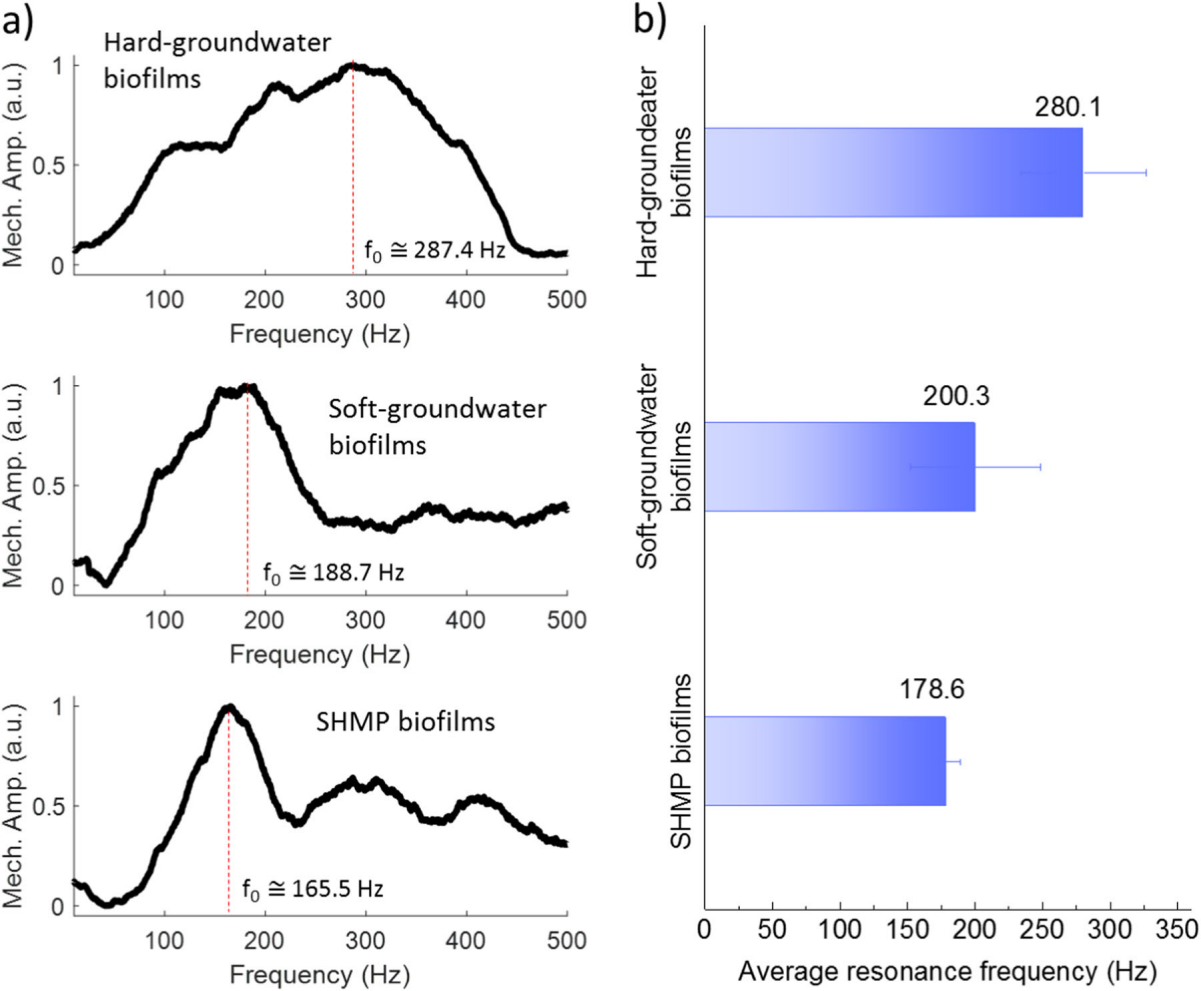
**a** The selected biofilm oscillation amplitude curves across a frequency ranged from 10 to 500 Hz. The peak in each curve represents the mechanical resonance frequency of each biofilm. **b** The average resonance frequency and standard deviation for each biofilm sample. The error bars represent the standard deviation of the measurements for the replicates

MM-OCE determined the biofilm resonance frequency, which reflects the biofilm overall stiffness in this study. The mechanical spectrum, showing the responding oscillation displacement (mechanical amplitude) across a chirped frequency range of 10–500 Hz, is plotted in Fig. 5a for the hard-groundwater, soft-groundwater, and SHMP biofilms. The frequency that corresponded to the maximum displacement amplitude in the mechanical spectrum was identified as the resonant frequency of the sample. For example, a peak at ~287.4 Hz, representing the resonance frequency, was observed in the mechanical spectrum of hard-groundwater biofilms. By measuring the mechanical spectrums in 12 different locations in the hard-groundwater biofilms, the mean resonance frequency of 280.1 Hz was obtained (Fig. 5b). Compared with the hard-groundwater biofilms, the soft-groundwater and SHMP biofilms showed a lower resonance frequency, where an average resonant frequency of 200.3 Hz and 178.6 Hz was observed, respectively. Based on the MM-OCE results, it is suggested that both the soft-groundwater and the SHMP biofilms have a lower overall stiffness as compared to the hard-groundwater biofilms.

## Discussion

We hypothesize that divalent ions and polyphosphate play an important role in biofilm stiffness. To test this hypothesis, we developed a methodology to effectively evaluate the effect of divalent ions and polyphosphate on biofilm physical and chemical properties. To the best of our knowledge, this is the first report revealing how the water chemistry influences the biofilm composition, structure, and stiffness and explores the possible link between biofilm properties. Specifically, groundwater containing a high divalent ion concentration (1.6 mM Ca^2+^ and 1.2 mM Mg^2+^) generated biofilms with a high content of CaCO_3_ and high stiffness; while the softened groundwater with a low divalent ion concentration (0.2 mM Ca^2+^ and 0.3 mM Mg^2+^) and groundwater containing SHMP produced biofilms with a low content of CaCO_3_ and low stiffness. The stiffer biofilms developed from hard groundwater would be expected to better resist flow shear stress, and thus have less detachment under water flow. On the contrary, the softer biofilms developed from soft groundwater and groundwater containing SHMP would be expected to detach more under flow shear stress.

The high stiffness of the hard-groundwater biofilms, as determined by the AFM and MM-OCE, was caused by the high content of CaCO_3_. Ca^2+^ in hard groundwater could bind with and cross-link the functional groups (e.g., carboxylate) in biofilm extracellular polymeric substances (EPS) or bacterial cells.^53,54^ The Ca^2+^ ions bound with EPS or bacterial cells can then form CaCO_3_ precipitates and lead to the integration of CaCO_3_ into the biofilm matrix. The existence of crystalline CaCO_3_, such as calcite and aragonite, in the hard-groundwater biofilms was revealed by ATR-FTIR. These CaCO_3_ crystals could fill the biofilm porous structure (the porosity is highly affected by EPS^55^) and thus make the biofilms more compact and stiffer. To prove this speculation, we imaged the hard-groundwater biofilms (high CaCO_3_ content) and SHMP biofilms (no CaCO_3_ content) by the environmental scanning electron microscope (ESEM). The hard-groundwater biofilms (Figure S3a) were less porous than the SHMP biofilms (Figure S3b), suggesting a more compact structure of hard-groundwater biofilms. Moreover, we obtained the elemental distribution map using the energy-dispersive spectroscopy (EDS) analysis for the hard-groundwater biofilms during ESEM imaging (Figure S4). The results showed that the Ca element was distributed across the whole biofilm, which further indicated that Ca was integrated with biofilms. Furthermore, while the CaCO_3_ crystal has a high stiffness (e.g., calcite elastic modulus ranges from 56 to 144 GPa),^56^ the presence of CaCO_3_ crystals integrated with the biomass could cause larger resistance to the indented probe or MNP-induced motion during the AFM or MM-OCE measurements. Therefore, the presence of CaCO_3_ in the hard-groundwater biofilms leads to the high stiffness measurement results.

The high content of CaCO_3_ in hard-groundwater biofilms was caused by the precipitation of CaCO_3_ formed from the high concentration of Ca^2+^ in groundwater. Biofilms and some bacteria were previously found to induce Ca^2+^ to precipitate as CaCO_3_.^57–59^ The precipitated CaCO_3_ can accumulate inside the biofilms and provide support to build up the biofilm structure.^60^ On the contrary, when the Ca^2+^ concentration was reduced in the soft groundwater, less CaCO_3_ precipitation and accumulation in biofilms would be expected, and thus little inorganic precipitates were detected in the soft-groundwater biofilms. When SHMP was added into the groundwater, the SHMP prevented CaCO_3_ precipitation by disturbing the formation of crystal structures. Specifically, SHMP could adsorb on the active growth sites of the CaCO_3_ and thus reduce the CaCO_3_ nucleation and crystal growth rates.^61^ Therefore, although the calcium element was detected in SHMP biofilms by ICP-MS, no precipitated CaCO_3_ was observed by ATR-FTIR.

Compared to the soft-groundwater and SHMP biofilms, higher near-surface stiffness and higher bulk stiffness of hard-groundwater biofilms were revealed by AFM and MM-OCE, respectively. The AFM indentation method was previously used to detect the elastic modulus of single culture biofilms (ranged from 0.1 to 8.6 kPa)^30,32,33^ and drinking water biofilms (ranged from 0.3 kPa to 9 MPa).^31,62^ To complement the AFM method that detects the stiffness of the top 2-μm surface layer, the MM-OCE method was used to detect the average biofilm bulk stiffness along the whole biofilm depth with a good spatial resolution (~6 µm in axial direction and ~16 µm in transverse direction) and with a short detection time (~2 s per chirped measurement). MM-OCE was previously used to determine the mechanical properties of biological tissues.^39,43^ Similar to biofilms, biological tissues often exhibit heterogeneity. MM-OCE, with its high spatial resolution (micrometer level) and displacement sensitivity (nanometer level), has been actively investigated and has shown potential in resolving the mechanical properties of biological tissues with high heterogeneity or with small dimensions.^63,64^ However, it should be noted that a precise measurement of localized mechanical properties is challenging in MM-OCE, as regions with different viscoelastic properties and geometries are mechanically coupled within the tissue in nature.^43^ In both tissues and biofilms, the bulk mechanical response can be assessed, while ongoing efforts are still being made for the evaluation of local elastic heterogeneities. This is the first study showing the potential of using MM-OCE on in situ biofilm bulk stiffness determination in environmental engineering.

Although both soft-groundwater and SHMP biofilms were soft and expected to be less resistant to flow shear stress, the SHMP biofilms were much thicker than soft-groundwater biofilms and thus would detach more under flow shear stress and raise more concerns of microbial safety in water. The higher thickness of SHMP biofilms was caused by the addition of the phosphorus nutrient during biofilm development. The observation that phosphorus promoting the accumulation of biofilm biomass was also reported in previous studies.^65–67^ Phosphorus can facilitate the bacterial growth and survival.^68,69^ Specifically, phosphorus is an important constituent of biomolecules such as DNA, RNA, and ATP. Phosphorus also takes an important role in energy delivery and regulation of cellular physiology.^68^ A low level of phosphorus in previous studies led to loss of cell fimbriae and less accumulation of biofilm biomass.^65,67^ Therefore, the biofilms developed from groundwater containing SHMP were much thicker than the biofilms developed from hard- and soft groundwater.

The results of this study suggested that adjustment of divalent ions and addition of polyphosphate influenced the biofilm physical and chemical properties. These findings provided insights on water quality improvement and engineered water system management. Our study showed that reducing divalent ion concentrations and applying polyphosphate could effectively control the accumulation of mineral deposits. Moreover, the soft biofilms generated from softened water and water containing polyphosphate would be easier to remove by hydraulic flushing. However, the thick and soft biofilms developed in the presence of SHMP may cause the lease of microorganisms and thus deteriorate the water quality.^15,16^ Especially, when SHMP was applied in water treatment infrastructures (e.g., filtration membrane) and drinking water distribution systems,^24,70^ the possible risk of biofilm-associated pathogen release could not be neglected. The results of this study on simulated drinking water biofilms could also be extended to the biofilm control and management of other engineered systems, including the food industries and medical devices. Because the results of this study suggested that common practice in the United States of adjusting divalent ions and adding polyphosphate for scale control could effectively influence the biofilm physical and chemical properties, future studies should focus on strategy development to optimize and balance the system maintenance and microbial contamination.

## Methods

### Biofilm preparation

Biofilms used in this study were developed from groundwater in Urbana-Champaign, IL (the local drinking water source). After filtration by a greensand filter, the main metal components in this groundwater included 1.65 ± 0.08 mM Ca^2+^, 1.16 ± 0.01 mM Mg^2+^, and 1.04 ± 0.02 mM Na^+^. The hardness, which was mainly contributed by Ca^2+^ and Mg^2+^, of this groundwater source was 281 ± 8 mg/L (the calculation of water hardness was described in SI). The total organic carbon (TOC) in this groundwater was 1–1.6 mg/L and the pH was 7.5–7.8. Three types of biofilms were developed from the raw groundwater with high hardness, groundwater with reduced hardness (or softened groundwater), and groundwater containing SHMP (Sigma-Aldrich), respectively. The groundwater with reduced hardness was prepared by mixing 10 L of groundwater with 20 g of Na^+^ from ion exchange resins (Amberlite® IR120 Na^+^ from Sigma-Aldrich) and stirring the mixture overnight. These Na^+^ compounds form ion exchange resins targeted on the cations (e.g., Ca^2+^, Mg^2+^, Fe^3+^, and Mn^2+^) in groundwater, but would not be expected to remove other anions and nonionic compounds from water. The ion exchange resins were separated from the water phase by settling down under quiescent conditions for 1 h and carefully transferring the supernatant to a new container. The ion exchange resin treatment reduced groundwater hardness to 49 ± 1 mg/L (0.16 mM Ca^2+^ and 0.34 mM Mg^2+^). According to the classification recommended by World Health Organization,^71^ the treated groundwater containing hardness of 49 ± 1 mg/L was considered to be soft (hardness < 60 mg/L), while the raw groundwater containing hardness of 281 ± 8 mg/L was very hard (hardness >180 mg/L). To prepare the groundwater containing SHMP, SHMP was added to groundwater to reach a final concentration of 10 ppm. SHMP is a widely used polyphosphate for scale control in engineered water systems.^72^ The maximal polyphosphate use level recommended by the National Sanitation Foundation (NSF) (NSF/ANSI 60–2013) is 11.9 ppm. The biofilms were developed on PVC coupons from those three types of water sources using CDC reactors, as described previously.^13,73^ Biofilm development took place under a shearing condition by continuously stirring the CDC reactors at 125 rpm. During biofilm development, no additional bacterial cultures or nutrients were added to the CDC reactors. Biofilms were allowed to develop in CDC reactors for 10 months before subsequent composition, structure, and stiffness determination.

### Biofilm composition

ATR-FTIR (PerkinElmer Inc., Waltham, MA) was used to examine the composition of hard-groundwater biofilms, soft-groundwater biofilms, and SHMP biofilms. Before each ATR-FTIR measurement, the biofilm coupons were carefully removed from the CDC reactors and rinsed in deionized water to remove the trace dissolved or suspended CaCO_3_ in water within the porous biofilm structure. After that, the biofilms were scratched from the coupon substrate and air-dried. The dried biofilms were then transferred to the ATR-FTIR crystal, and the absorbance was measured in the wavenumber range from 400 to 4000 cm^−1^. For each type of biofilms, three biofilm coupons were chosen for infrared spectroscopy measurements, and 8–16 scans were obtained for each sample. The different frequencies of molecular vibrations served to identify the biofilm composition by comparing these to databases and previous studies.^28,46–48,60,74^

In addition to spectroscopy, TGA (PerkinElmer Inc., Waltham, MA) was also applied for biofilm composition determination. Different biofilm components are pyrolyzed at different temperatures. TGA can quantify the weight loss of biofilms caused by pyrolysis as a function of elevating temperature. Before TGA measurements, the biofilms were scratched from the PVC coupon substrate and dehydrated by incubating at 37 °C for 4 h. The initial weight of the dehydrated biofilms was determined after transferring the biofilms to the TGA pan. During each TGA measurement, the change of biofilm weight over a temperature range from 30 to 800 °C at a heating rate of 10 °C/min was monitored. The percentage of residual biofilm mass, determined as the biofilm weight at a certain temperature divided by the initial biofilm weight, as a function of temperature was determined accordingly. N_2_, at a rate of 20 ml/min, was used as purge gas in all the TGA measurements. Both ATR-FTIR and TGA methods are required to remove biofilms by scratching before measurements. However, the process of removing biofilms from PVC coupons would not change the original biofilm chemical composition.

### Biofilm structure determination using OCT

Biofilm thickness and roughness were determined by OCT, according to previous studies.^13,26^ The cross-sections of biofilms were imaged by a custom-built 1300-nm-based spectral domain OCT system with an axial resolution of ~6 µm and a transverse resolution of ~16 µm. A mode-locked titanium sapphire laser source (Kapteyn-Murnane Laboratories, Inc., Boulder, CO) was used in the OCT system to acquire images with a size of 3.1 by 2.1 mm. The biofilm coupons were kept in water during transfer and the imaging process. For each type of biofilm, 900 images for nine locations in three biofilm coupons were obtained by OCT. One hundred and thirty-five images for each type of biofilms were selected randomly for further biofilm thickness and roughness analysis.^13,31,73^ Briefly, ImageJ (http://imagej.nih.gov/ij/) was used to eliminate the background of selected images. After that, a previously developed MATLAB program was used to quantify the biofilm thickness and roughness.^75^ The program detected the PVC–biofilm interface, binarized the image by grayscale gradient analysis and automatic thresholding, and calculated the values of the local biofilm thickness (*z*_*i*_ in µm) at each location in each image. Then the mean biofilm thickness ($\bar z$ in µm), absolute biofilm roughness (*R*_*a*_), and relative roughness (*R*_*a*_*’*) coefficients were calculated according to the following equations:

1 $$\bar z = \frac{1}{n}\mathop {\sum }\limits_{i = 1}^N Z_i$$

2 $$R_a = \frac{1}{n}\mathop {\sum }\limits_{i = 1}^N \left( {\left| {Z_i - \bar Z} \right|} \right)$$

3 $$R_a = \frac{1}{n}\mathop {\sum }\limits_{i = 1}^N \left( {\frac{{\left| {Z_i - \bar Z} \right|}}{Z}} \right)$$

where *N* is the number of thickness measurements in each image.

### Biofilm stiffness determination using AFM indentation

AFM indentation tests were applied to determine the biofilm near-surface elastic modulus, as described previously.^31^ The colloidal probe used in indentation tests was prepared by adhering a silica sphere (with a diameter of 20 µm) to a tipless cantilever (calibrated spring constant=0.7 N/m, Mikromasch, Lady’s Island, SC). To determine the colloidal probe cantilever deflection sensitivity, the probe was calibrated before the indentation tests by obtaining the force curves on a piece of glass slide both in air and in water.^76^ All the indentation tests were conducted under a contact mode by a MFP-3D AFM (Asylum Research, Santa Barbara, CA). The tested biofilm coupons were maintained in water before subjecting to the indentation tests. During the indentation tests, the indentation force as a function of indentation depth into the biofilms was obtained. To avoid substrate effects, the maximal indentation depths in all indentation tests were smaller than 3 µm;^77,78^ thus, only the near-surface region was probed. To maintain the biofilm mechanical properties, all the indentation tests were conducted in liquid (sterilized DI water). The speed was maintained constant at a rate of 2 μm/s. Hertz model was used to fit the indentation curves and obtain the biofilm elastic modulus.^76^ More details on AFM indentation tests and indentation curve analysis were described in our previous study.^31^ For each type of biofilms, 30 locations in three biofilm coupons were randomly selected for indentation tests. At each location, 10–20 replicates of indentation curves were acquired. Since the biofilms are heterogamous, the frequency distribution of the elastic modulus values measured at different locations was obtained for each type of biofilm. Kolmogorov–Smirnov test was used to compare the elastic modulus frequency distributions of different types of biofilms.

### Biofilm stiffness determination using MM-OCE

The MM-OCE system is composed of an OCT imaging system and a magnetic field generator (Figure S1, Supplementary Information). MNPs were introduced into the examined materials to serve as internal force transducers, which can be modulated by an external magnetic field to induce motion in the MNP-laden materials. Previous study showed a good penetration of MNPs in chicken breast tissues to at least ~2.2 mm in depth by using a magnetomotive-OCT (MM-OCT).^40^ Therefore, it is believed that MNPs can penetrate the 30–418-μm biofilms that are more porous and thinner than chicken breast. The magnetically induced motion (termed “magnetomotion”) of the biofilm is then monitored by OCT. By modulating an alternating magnetic field with a sinusoidal waveform, an oscillating force at the modulated frequency can be exerted to the MNP-laden sample, and the responding displacement amplitude can be measured optically for the examined sample. During the measurement, the small MNPs only exert internal force loading in biofilms, without causing large inertia or any biomass loss. When the excitation frequency is equal to the resonant frequency of the examined material, the oscillation amplitude achieves a maximum. The resonance frequency reflects the material stiffness and, with known sample geometry and mass density, can be converted into an elastic modulus. MM-OCE probes the biofilm bulk resonance frequency based on the temporally resolved resonant frequency, instead of the absolute displacement amplitude at each localized point. ^39–44,79^ Therefore, although the MNPs may distribute nonuniformly along the depth of biofilm bulk, these potential MNP concentration gradients are likely to have a negligible influence on the resonance frequency measurement results. MM-OCE can provide a higher resolution and requires lower detection time, compared with other elastography techniques (e.g., magnetic resonance and ultrasound elastography).

Before MM-OCE measurements, the MNP suspension was prepared by suspending magnetic iron (II,III) oxide (Fe_3_O_4_) particles (50–100-nm diameter, Sigma-Aldrich) in water to reach a final concentration of 2 mg/ml. To introduce the MNPs into the examined biofilms, the biofilm coupon, which was fixed by a plastic ring, was immersed into MNP suspension in a 50-ml centrifuge tube and slowly rotated at 7 rpm by a tube disk rotator. After that, the biofilm coupon was carefully removed from the plastic ring and rinsed with DI water three times right before the stiffness measurements. During the stiffness measurements, a chirp excitation with a frequency ranging from 10 to 500 Hz was applied to the MNP-laden biofilm samples. A 1983-Hz line-scan rate was utilized for OCT to ensure sufficient sampling (which meets the Nyquist criteria) and to prevent excessive oversampling. The motion (or displacement) of the biofilms at each excitation frequency was measured by OCT and then converted into a mechanical amplitude. This amplitude was plotted as a function of the excitation frequency to obtain a frequency-swept mechanical spectrum (Fig. 5). For each type of biofilm, 9–12 locations in three biofilm coupons were selected for stiffness determination using MM-OCE measurements.

### Data availability statement

The data sets generated and analyzed during the current study are available in the figshare repository (DOI: 10.6084/m9.figshare.6344666). The raw OCT and ESEM images are available from the corresponding authors upon reasonable request.

## Electronic supplementary material

Supplementary Information
