# Supplementary material for: Effect of divalent ions and a polyphosphate on composition, structure, and stiffness of simulated drinking water biofilms
Source: NPJ Biofilms Microbiomes. 2018 Jul 18;4:15. doi: 10.1038/s41522-018-0058-1 (PMC6052100; doi:10.1038/s41522-018-0058-1)
Supplement: Supplementary file 1 — Supplementary Information [file 41522_2018_58_MOESM1_ESM.pdf]

Effect of divalent ions and a polyphosphate on composition, structure, and stiffness of simulated drinking water biofilms

Yun Shen[1*†], Pin Chieh Huang[2], Conghui Huang[1], Peng Sun[1], Guillermo L. Monroy[2], Wenjing Wu[1], Jie Lin[1], Rosa Espinosa-Marzal[1], Stephen A. Boppart[2,3], Wen-Tso Liu[1], Thanh H. Nguyen[1]

[1]Department of Civil and Environmental Engineering, University of Illinois at Urbana-Champaign, [2]Department of Bioengineering, University of Illinois at Urbana-Champaign, [3]Department of Electrical and Computer Engineering, University of Illinois at Urbana-Champaign.

Supplementary Information

2 PAGE
2 FIGURE

[*] Corresponding author contact information:
Yun Shen
Phone: 217 898 1087
Email: yunshen@umich.edu
[†] Corresponding author present address:
1351 Beal Ave., 219 EWRE Bldg.
University of Michigan
Ann Arbor, MI 48109-2125

Metallic element determination by inductively coupled plasma mass spectrometry (ICP-MS)

Inductively coupled plasma mass spectrometry (ICP-MS, PerkinElmer – SCIEX ELAN DRCe ICP-MS, Norwalk, CT USA) was used to measure the metallic elements of biofilm samples and reactor feed water. The ICP-MS method was described in US EPA SW-846 Test Method 6020B. Before measuring metallic elements of biofilm samples, the biofilms were digested in nitric acid (5% v/v) for two hours. Then, Ca, Mg, and Na contents were analyzed for the digested biofilm solutions and reactor feed water samples.

Groundwater hardness calculation

In the groundwater used in this study, Ca^{2+} and Mg^{2+} are the two dominant cations composed the majority of water hardness. Therefore, the overall water hardness is calculated by summing up the contributions from Ca^{2+} and Mg^{2+}, as shown below:

Step 1. Calculate water hardness contributed by Ca^{2+} (1.65 mM or 1.65×10^{-3} mol/L in our groundwater):

$$\left(1.65 \times 10^{-3}\ \frac{mol\ Ca^{2+}}{L}\right) \times \left(1\frac{mol\ CaCO_3}{mol\ Ca^{2+}}\right) \times \left(100\ \frac{g\ CaCO_3}{mol\ CaCO_3}\right) = 0.165\frac{g\ CaCO_3}{L}\ or\ 165\ mg\ CaCO_3/L$$

Step 2. Calculate water hardness contributed by Mg^{2+} (1.16 mM or 1.16×10^{-3} mol/L in our groundwater):

$$\left(1.16 \times 10^{-3}\ \frac{mol\ Mg^{2+}}{L}\right) \times \left(1\frac{mol\ CaCO_3}{mol\ Mg^{2+}}\right) \times \left(100\ \frac{g\ CaCO_3}{mol\ CaCO_3}\right) = 0.116\frac{g\ CaCO_3}{L}\ or\ 116\ mg\ CaCO_3/L$$

Step 3. Total hardness: 165+116=281 mg $CaCO_3$/L.

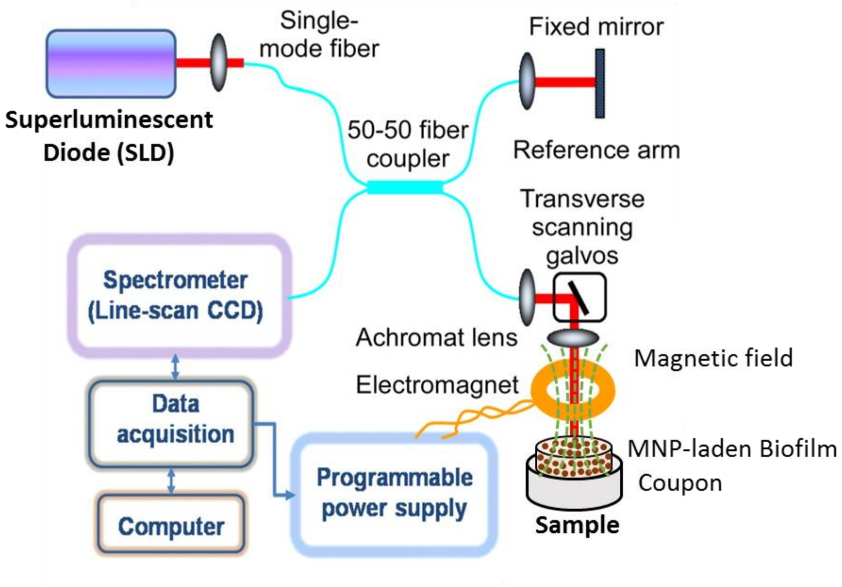

Figure S1 Configuration of the MM-OCE system. The MNPs inside biofilms oscillate and induce the motion of the biofilm components, driven by the magnetic field powered by a programmable power supply. The nanometer-scale motion of the biofilm can then be captured by the OCT imaging system. The biofilm stiffness can subsequently be determined by the biofilm motion and strength of the magnetic field. This figure was adapted from a previous MM-OCE study.[1]

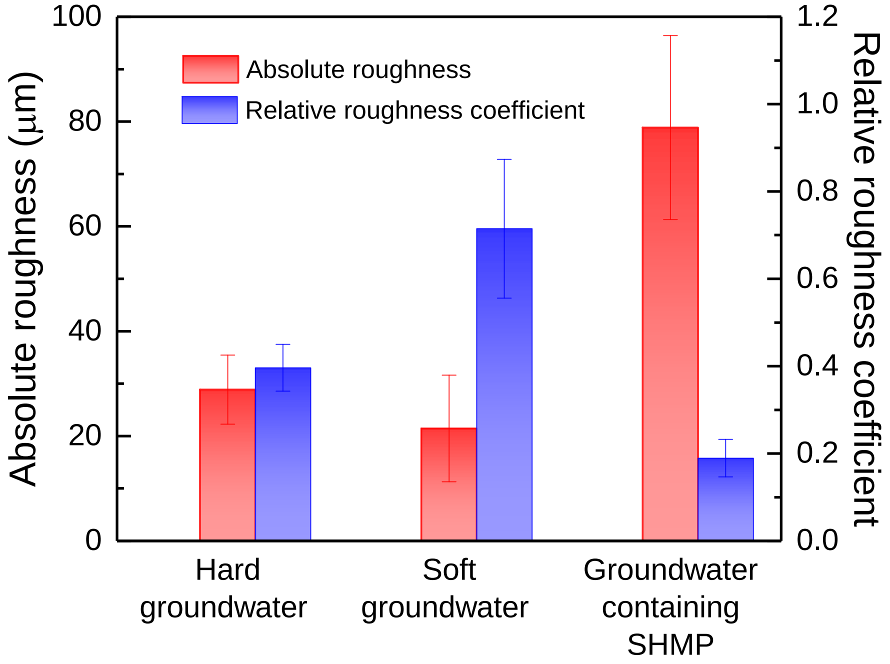

Figure S2 Absolute roughness and relative roughness coefficient of biofilms developed from hard-groundwater, soft-groundwater, and groundwater containing SHMP.

a) Hard-groundawater biofilms

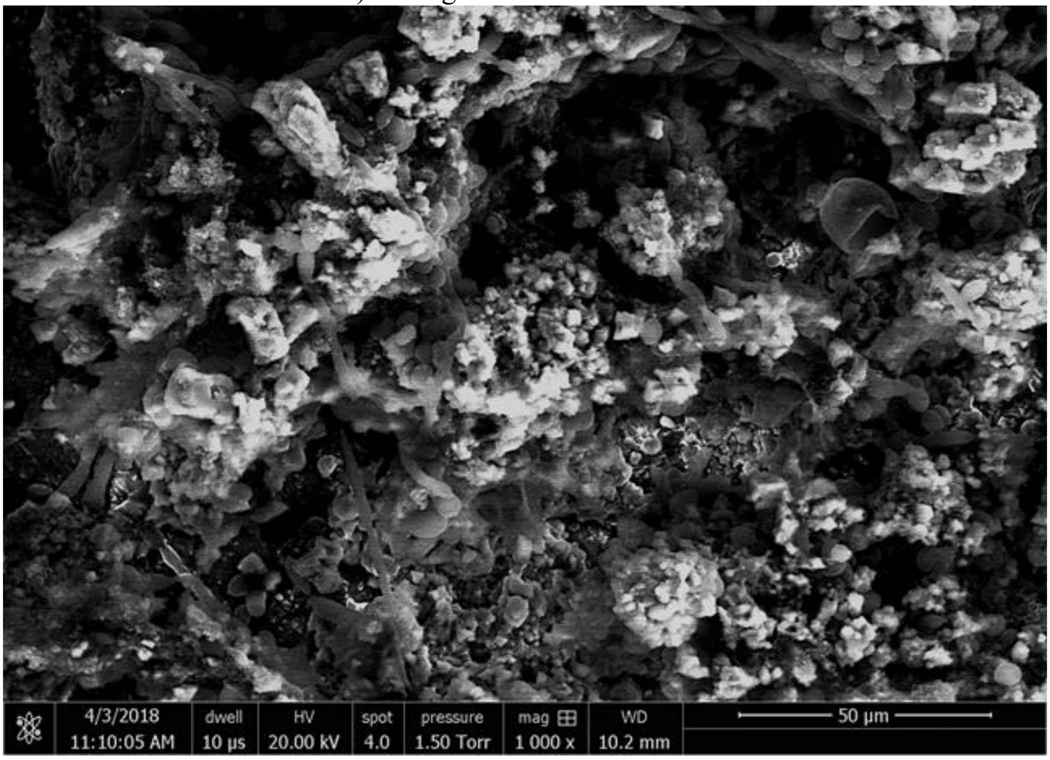

b) SHMP biofilms

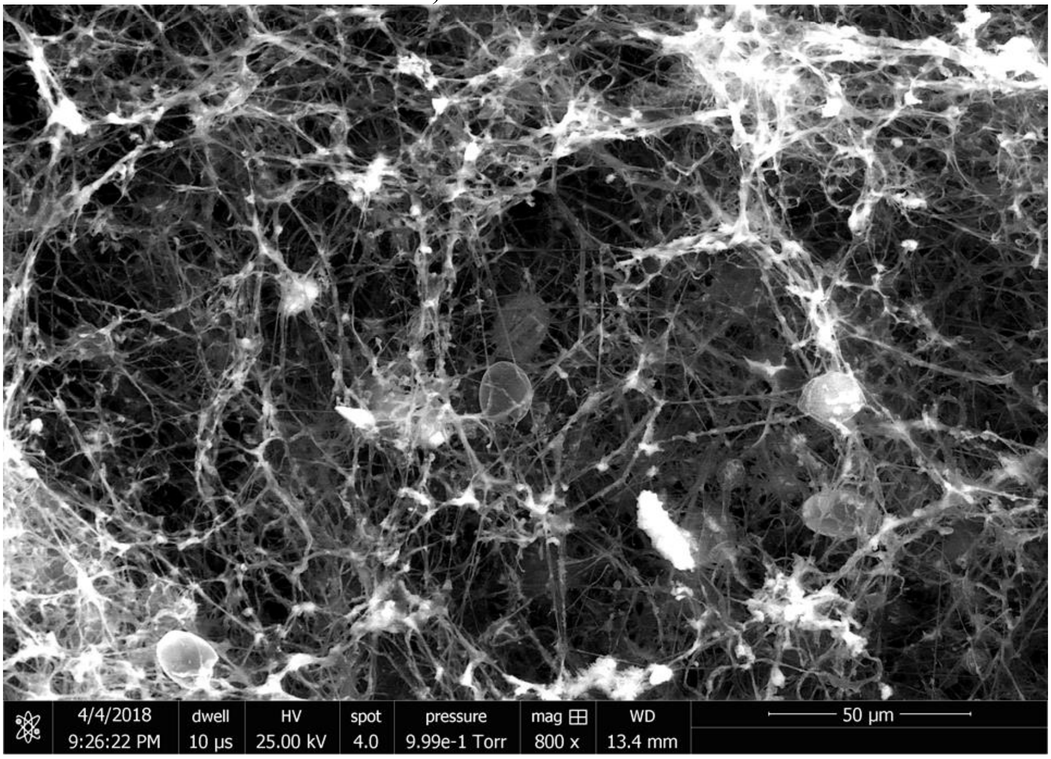

Figure S3 Environmental scanning electron microscope (ESEM) images of a) hard-groundwater biofilms and b) SHMP biofilms. The images were taken using a FEI Quanta FEG 450 ESEM (FEI Company). Before imaging, the biofilms were dried with a CO2 critical point dryer (Tousimis, MD) and were coated with gold-palladium. More details of biofilm preparation and ESEM imaging were described in our previous study.[2]

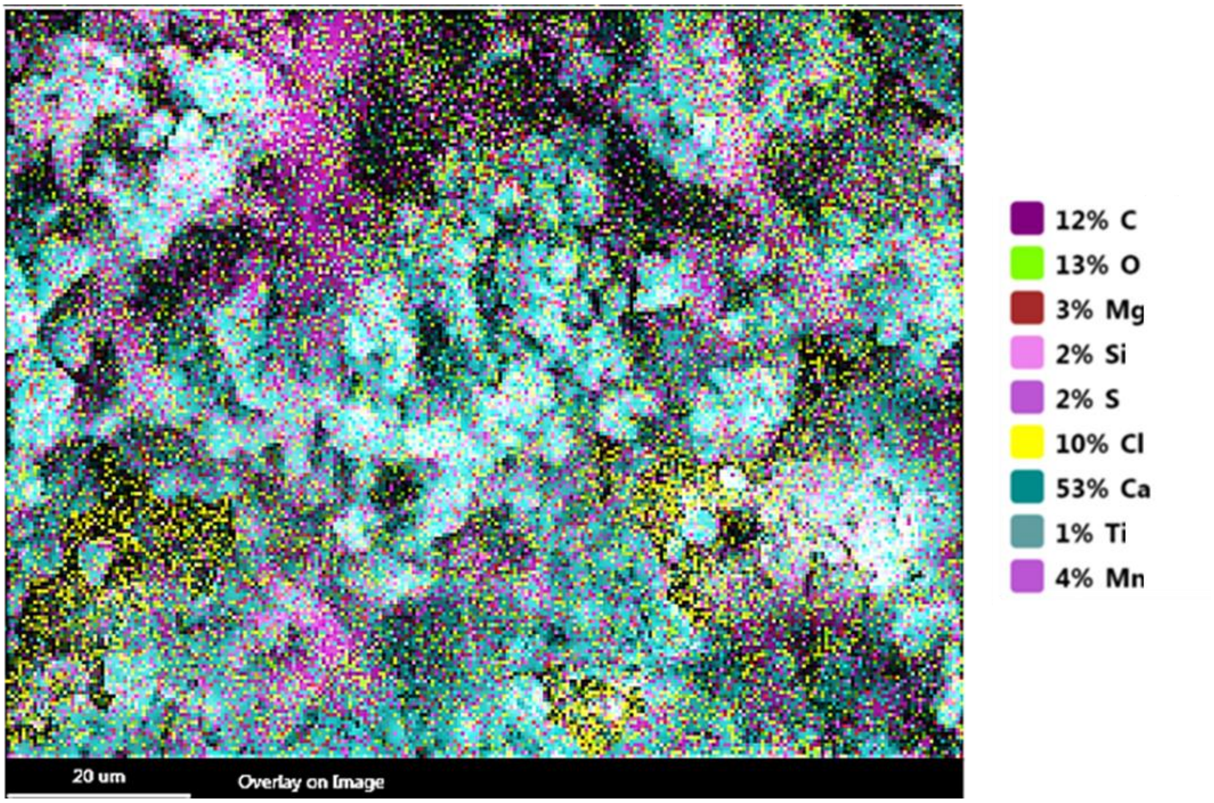

Figure S4 Elemental distribution map in the hard-groundwater biofilms. The element of C, O, Mg, Si, S, Cl, Ca, Ti, and Mn was measured by energy-dispersive spectroscopy (EDS) during ESEM imaging. The element distribution map overlaid with the ESEM image.

Table S1 Water quality parameters for the simulated drinking water used in this study

Water quality parameters	Concentrations
**Calcium (Ca)**	66.18 ± 2.95 ppm
**Magnesium (Mg)**	27.94 ± 0.23 ppm
**Sodium (Na)**	24.12 ± 0.62 ppm
**Iron (Fe)**	11.05 ±17.60 ppb
**Manganese (Mn)**	4.33 ±1.53 ppb
**Total organic carbon (TOC)**	1.3 ± 0.42 mg/L
**Nitrate nitrogen (NO_3-N)**	0.04 ± 0.02 mg/L
**Ammonia nitrogen (NH_3-N)**	0.01 ± 0.01 mg/L

References

1 John, R. *et al.* In vivo magnetomotive optical molecular imaging using targeted magnetic nanoprobes. *Proc. Natl. Acad. Sci.* **107**, 8085-8090, (2010).

2 Janjaroen, D. *et al.* Roles of ionic strength and biofilm roughness on adhesion kinetics of *Escherichia coli* onto groundwater biofilm grown on PVC surfaces. *Water Res* **47**, 2531-2542, (2013).
